# Delta Neutrophil Index as a Promising Prognostic Marker in Out of Hospital Cardiac Arrest

**DOI:** 10.1371/journal.pone.0120677

**Published:** 2015-03-23

**Authors:** Ho Young Yune, Sung Phil Chung, Yoo Seok Park, Hyun Soo Chung, Hye Sun Lee, Jong Wook Lee, Jong Woo Park, Je Sung You, Incheol Park, Hahn Shick Lee

**Affiliations:** 1 Department of Emergency Medicine, Yonsei University College of Medicine, Seoul, Republic of Korea; 2 Department of Research Affairs, Biostatistics Collaboration Unit, Yonsei University College of Medicine, Seoul, Republic of Korea; 3 Department of Laboratory Medicine, Jincheon Sungmo Hospital, Jincheon, Republic of Korea; 4 Department of Emergency Medicine, Changwon Fatima Hospital, Changwon, Republic of Korea; 5 Department of Emergency Medicine, Graduate School of Medicine, Kangwon National University, Chuncheon, Republic of Korea; Azienda Ospedaliero-Universitaria Careggi, ITALY

## Abstract

**Background:**

The post-resuscitation phase after out-of-hospital cardiac arrest (OHCA) is characterised by a systemic inflammatory response (e.g., severe sepsis), for which the immature granulocyte count is a diagnostic marker. In this study we evaluated the prognostic significance of the delta neutrophil index (DNI), which is the difference in leukocyte subfractions as assessed by an automated blood cell analyser, for early mortality after OHCA.

**Materials and Methods:**

OHCA records from the emergency department cardiac arrest registry were retrospectively analysed. Patients who survived at least 24 h after return of spontaneous circulation were included in the analysis. We evaluated mortality and cerebral performance category scores at 30 days.

**Results:**

A total of 83 patients with OHCA were included in the study. Our results showed that DNI >8.4% on day 1 (hazard ratio [HR], 3.227; 95% CI, 1.485–6.967; p = 0.001) and DNI >10.5% on day 2 (HR, 3.292; 95% CI, 1.662–6.519; p<0.001) were associated with increased 30-day mortality in patients with OHCA. Additionally, DNI >8.4% on day 1 (HR, 2.718; 95% CI, 1.508–4.899; p<0.001) and DNI >10.5% on day 2 (HR, 1.709; 95% CI, 1.051–2.778; p = 0.02) were associated with worse neurologic outcomes 30 days after OHCA.

**Conclusion:**

A higher DNI is a promising prognostic marker for 30-day mortality and neurologic outcomes after OHCA. Our findings indicate that patients with elevated DNI values after OHCA might be closely monitored so that appropriate treatment strategies can be implemented.

## Introduction

The incidence of out-of-hospital cardiac arrest (OHCA) in United States has increased from 295,000 cases in 2009 to approximately 424,000 cases in 2011.[1,2] In the early post-resuscitation phase after OHCA, the complex pathophysiological processes of post-cardiac arrest syndrome involve a systemic inflammatory response (e.g., severe sepsis).[3–6] Sepsis-related physiologic derangements are an important cause of early mortality in resuscitated patients.[4,5,6] Currently there are no widely accepted prognostic factors to predict the severity of sepsis or mortality in resuscitated patients during this early critical period.

Immature granulocytes are an indicator of increased myeloid cell production and are associated with infection or systemic inflammation.[7–10] Although the immature granulocyte count is a marker for septic conditions, this measure is difficult to use in clinical practice because manual counting is not accurate.[7,8] Recently, Nahm et al. developed the delta neutrophil index (DNI), which is the difference in leukocyte subfractions as assessed by an automated blood cell analyser.[9] This method determines the fraction of circulating immature granulocyte as the difference between the leukocyte subfraction determined by the cytochemical myeloperoxidase reaction and the leukocyte subfraction determined in a nuclear lobularity assay by the reflected light beam.[9,10]

Recent studies suggest that the DNI is associated with positive blood culture results, disseminated intravascular coagulation, and mortality in critically ill patients with suspected sepsis.[8,9,10] Because patients resuscitated after cardiac arrest experience post-cardiac arrest syndrome, which includes sepsis-like physiologic derangement, the DNI may be associated with early mortality or neurologic outcomes. Therefore, in this study we evaluated DNI values of patients resuscitated after cardiac arrest to determine the prognostic significance of DNI as a marker for early mortality after OHCA.

## Materials and Methods

This study was approved by the institutional review board of Yonsei University College of Medicine, Gangnam Severance Hospital and performed between March 2010 and November 2012 at an urban hospital affiliated with our institution. Patients’ records and information were anonymized and de-identified prior to analysis as retrospective study. This hospital has an emergency department census of 60,000 patients per year. We retrospectively analysed OHCA records from the emergency department cardiac arrest registry, screening all patients who experienced return of spontaneous circulation (ROSC) after OHCA. Those who survived at least 24 h after ROSC were included in the analysis. Exclusion criteria were age <18 years, traumatic OHCA, and OHCA related to a drug overdose, hanging, or asphyxia. Management of patients with OHCA was based on the 2010 European resuscitation council/American Heart Association guidelines.

Upon arrival at the emergency department, all patients immediately had blood drawn for the routine blood sampling set and were managed according to the resuscitation protocol of our institution. We extracted traditional Utstein template data from the emergency department cardiac arrest registry including age, sex, patient identifier, the underlying significant co-morbidities (hypertension, diabetic mellitus, pulmonary disease, malignancy, cardiovascular disease, renal disease, the presence of a prior acute coronary syndrome,), date of arrest, location of arrest (public location or not), witnessed, bystander cardiopulmonary resuscitation (CPR), prehospital ROSC, emergency medical services (ambulance or not), first monitored rhythm (shockable or nonshockable), the use of antibiotic prophylaxis and inotrophics, the Acute Physiology and Chronic Health Evaluation II (APACHE II) score on admission, therapeutic hypothermia, survived event, pre-hospital ROSC, end of event, and cause of arrest. Serum DNI was measured on day 1 (blood drawn immediately on emergency department admission), day 2 (24 h after admission), and day 3 (48 h after admission), and peak DNI (highest DNI value during the hospital stay) was determined. Other laboratory tests including cell count, blood urea nitrogen, creatinine, red cell distribution width, and albumin were carried out using the initial blood specimens collected at emergency department admission.

DNI was automatically determined by an automated blood cell analyser (ADVIA 2120, Siemens, Forchheim, Germany) using the following formula: DNI = (neutrophil subfraction and eosinophil subfraction measured in the myeloperoxidase channel)–(polymorphonuclear subfraction measured in the nuclear lobularity channel) [9,10] Post-resuscitation care was performed in accordance with the 2005 and 2010 American Heart Association Guidelines for Cardiopulmonary Resuscitation and Emergency Cardiovascular Care.

Clinical outcomes were mortality and cerebral performance category (CPC) score at 30 days. CPC scores of 1 or 2 indicated favourable outcomes, whereas CPC scores of 3 to 5 indicated poor outcomes; a CPC score of 5 was assigned to patients who remained comatose and died during the hospital stay or within 30 days after OHCA. Patients who were discharged or transferred to other hospital were interviewed by telephone within 30 days after OHCA.

### Statistical analysis

Demographic and clinical data are presented as median (range) or frequency, as appropriate. Continuous variables were compared by independent two-sample t-test, and categorical variables were compared by chi-squared test or Fisher exact test. Univariate analysis was carried to evaluate relationships among demographic characteristics, Utstein variables, 30-day mortality, and 30-day CPC score. Multivariate Cox proportional hazard regression analysis was conducted to identify independent prognostic factors, and results were expressed as hazard ratios (HRs) and 95% confidential intervals (CIs). We selected age and sex as known clinically important variables. To highlight independent indicators of outcomes, we used a multivariate Cox proportional hazard regression analysis integrating major covariates (selected here as variables with a *P* < 0.1) indicated from our univariate analysis. In our study, we analyzed initial rhythm as a significant variable (*P* < 0.1) together with age and sex. Mean DNI values were compared between patient groups with respect to 30-day mortality (surviving versus non-surviving) and neurologic outcomes (good versus poor).

Kaplan–Meier analysis survival curves were generated based on 30-day mortality and 30-day CPC scores, and the groups were compared by the log-rank test. Previous studies about DNI in sepsis simply predicted cut-off points based on only events.[7–11] However, our study made careful effort to predict the optimal cut-off point based on time to events through by using the Contal and O’Quigley technique. This technique was used to select the cut-off value of DNI for dichotomization of outcome variables. The optimal cut-off point was selected by maximizing the hazard ratio.[12] A post-hoc statistical power calculation was performed using a hazard ratio assumed to be α = 0.05; we had 86% and 87% post-hoc power to detect a hazard ratio (3.227, 3.292) of DNI1, DNI2 aspect to mortality. Statistical analyses were carried out using SAS version 9.2 (SAS Institute Inc., Cary, NC); p<0.05 was considered significant.

## Results

Of the 517 patients with OHCA registered in the emergency department cardiac arrest registry during the study period, sustained ROSC was achieved in 183 (35.4%) patients. A total of 101 (19.5%) patients survived at least 24 after ROSC. However, 18 patients were excluded from the analysis because of age <18 years (n = 9), traumatic OHCA (n = 8), or OHCA related to hanging (n = 1). The remaining 83 (16.1%) eligible patients were stratified according to mortality and neurologic outcomes at 30 days (Table 1). Thirty-four of the 83 subjects (41.0%) survived, and 12 (14.5%) showed good outcomes (CPC score 1 or 2) at 30 days after OHCA. DNI values on day 1, 2, and 3 and peak DNI differed significantly between the two patient groups.

**Table 1:** Baseline characteristics of patients stratified according to 30-day mortality and neurologic outcomes.

Variables	Total	30-day Mortality	30-day CPC score		30-day CPC score		
		Death (n = 49)	Survival (n = 34)	p-value	Good (n = 12)	Poor (n = 71)	p-value
**Age, years**	61.4±15.9	59.2±17.2	64.6±13.5	0.13	45±18.0	64.2±13.8	<0.001*
**Sex (Female)**	20 (23.1)	10 (20.4)	10 (29.4)		3 (25.0)	17 (23.9)	>0.999
**Hypertension**	36 (43.4)	22 (44.9)	14 (41.2)	0.737	3 (25.0)	33 (46.5)	0.165
**Diabetic mellitus**	24 (28.9)	12 (24.5)	12 (35.3)	0.286	2 (16.7)	22 (31.0)	0.494
**Pulmonary disease**	3 (3.6)	3 (6.1)	0 (0.0)	0.266	1 (8.3)	2 (2.8)	0.378
**Malignancy**	4 (4.8)	2 (4.1)	2 (5.9)	>0.999	0 (0.0)	4 (5.6)	>0.999
**Cardiovascular disease**	20 (24.1)	13 (26.5)	7 (20.6)	0.534	3 (25)	17 (23.9)	>0.999
**Previous ACS**	7 (8.4)	5 (10.2)	2 (5.9) 0	0.695	1 (8.3)	6 (8.5)	>0.999
**Renal disease**	13 (15.7)	9 (18.4)	4 (11.8)	0.416	0 (0.0)	13 (18.3)	0.198
**Cardiac origin**	47 (56.6)	30 (61.2)	17 (50.0)	0.31	10 (83.3)	37 (52.1)	0.444
**Cardiac arrest in public place**	35 (42.7)	22 (44.9)	13 (39.4)	0.621	4 (33.3)	31 (44.3)	0.478
**Pre-hospital ROSC**	1 (1.2)	1 (2.0)	0 (0.0)	>0.999	1 (8.3)	0 (0.0)	0.145
**Initial rhythm shockable**	22 (26.5)	17 (34.7)	5 (14.7)	0.043*	8 (66.7)	14 (19.7)	0.002*
**Total CPR time in hospital, min**	13.5±11.2	12.8±10.5	14.5±12.3	0.496	14.3±12.9	13.3±11.0	0.793
**Arrest time, min**	31.0±15.6	29.3±14.7	33.4±16.9	0.243	35.1±15.9	30.3±15.6	0.323
**Bystander CPR**	19 (23.5)	10 (20.8)	9 (27.3)	0.502	3 (25.0)	16 (23.2)	>0.999
**Hypothermia**	44 (53.0)	27 (55.1)	17 (50.0)	0.647	6 (50.0)	38 (53.5)	0.821
**Witness**	31 (37.35)	17 (34.69)	14 (41.18)	0.548	4 (33.33)	27 (38.03)	>0.999
**BUN, mg/dL**	26.6±19.2	28.0±22.4	24.6±13.1	0.39	21.9±22.0	27.4±18.7	0.36
**Cr, mg/dL**	2.6±3.2	2.9±3.7	2.2±2.2	0.244	1.4±0.7	2.8±3.4	0.002*
**Albumin, g/dL**	3.4±0.7	3.5±0.6	3.3±0.9	0.296	3.6±0.8	3.4±0.7	0.393
**Platelet count**	139.5±64.9	173.0±53.3	109.8±61.7	0.04*	152.3±67.4	135.6±66.4	0.668
**Haematocrit, %**	40.3±9.3	40.4±9.3	40.1±9.4	0.905	43.3±13.1	39.8±8.5	0.387
**RDW day 1, %**	13.9±1.6	13.8±1.6	14.1±1.5	0.285	13.2±1.0	14.0±1.6	0.087
**WBC day 1**	14,841.3±7642.2	16,678.6±8255.9	12,193.5±5809.1	0.005*	16,085.8±8692.0	14,631.0±7498.8	0.545
**DNI day 1, %**	5.3±9.1	3.6±5.1	7.7±12.5	0.076	2.3±2.9	5.8±9.6	0.02*
**DNI day 2, %**	10.7±12.8	7.5±7.9	15.3±16.8	0.015*	7.0±6.8	11.3±13.5	0.099
**DNI day 3, %**	8.3±9.7	5.3±6.3	15.3±12.5	0.003*	4.3±3.9	9.2±10.4	0.009*
**DNI day peak, (%)**	14.3±14.4	10.9±11.1	20.8±17.7	0.017*	8.0±6.4	15.7±15.3	0.008*
**DNI peak, day (day)**	3.1±3.7	2.5±2.0	4.2±5.6	0.17	2.7±3.0	3.2±3.9	0.635
**Antibiotics prophylaxis**	50 (82.0)	41 (85.4)	9 (69.2)	0.226	11(91.7)	39 (79.6)	0.438
**Inotrophics**	44 (72.1)	35 (72.9)	9 (69.2)	>0.999	8 (66.7)	36 (73.5)	0.723
**APACHE II**	19.0±5.7	18.7±6.3	19.5±4.7	0.508	17.1±7.4	19.3±5.3	0.209

CPC: cerebral performance category, ACS: acute coronary syndrome, ROSC: return of spontaneous circulation, CPR: cardiopulmonary resuscitation, BUN: blood urea nitrogen, Cr: creatinine, RDW: red cell distribution width, APACHE II:Acute Physiology and Chronic Health Evaluation II, DNI: delta neutrophil index

*: *p*<0.05.

The univariate Cox proportional hazard model revealed that higher DNI values on days 1, 2, and 3 were strong risk factors for mortality and poor neurologic outcomes at 30 days after OHCA (Table 2). The multivariate Cox proportional hazard model also showed that increased DNI values on day 1, 2, and 3 and peak DNI were independent predictors of mortality 30 days after OHCA, and DNI values on days 1 and 2 were independent predictors of poor neurologic outcomes (Table 3).

**Table 2:** Univariate Cox proportional analysis for 30-day mortality and neurologic outcomes.

Variables		30-day Mortality			30-day CPC score	
	HR	95% CI	p-value	HR	95% CI	p-value
**Age**	1.028	1.005–1.054	0.016*	1.028	1.012–1.045	<0.001^*^
**Sex (Female)**	1.235	0.589–2.587	0.577	0.895	0.518–1.547	0.691
**Hypertension**	1.069	0.539–20123	0.8479	1.303	0.815–2.082	0.2691
**Diabetic mellitus**	1.42	0.697–2.889	0.3339	1.286	0.773–2.140	0.3326
**Pulmonary disease**	0.443	0.025–7.791	0.5782	0.782	0.191–3.204	0.7323
**Malignancy**	1.507	0.359–6.324	0.575	1.622	0.588–4.479	0.3504
**Cardiovascular disease**	0.837	0.364–1.924	0.6751	1.041	0.603–1.798	0.8842
**Previous ACS**	0.635	0.151–2.661	0.534	1.033	0.444–2.404	0.9403
**Renal disease**	0.573	0.201–1.630	0.2964	0.868	0.473–1.592	0.6478
**Cardiac origin**	0.748	0.381–1.469	0.3996	0.8/76	0.548–1.399	0.5788
**Cardiac arrest in public place**	0.969	0.48–1.955	0.93	1.254	0.781–2.015	0.349
**Prehospital ROSC**	2.068	0.114–37.356	0.623	2.068	0.114–37.356	0.623
**CPR rhythm (Shockable)**	0.414	0.16–1.07	0.069	0.574	0.32–1.031	0.063
**Total CPR time in hospital**	1.009	0.981–1.038	0.533	1.003	0.982–1.024	0.793
**Arrest time**	1.014	0.993–1.036	0.188	1.003	0.989–1.018	0.646
**Bystander CPR**	1.719	0.793–3.727	0.17	1.461	0.831–2.567	0.188
**Hypothermia**	0.741	0.378–1.455	0.384	0.809	0.506–1.292	0.374
**Witness**	1.338	0.674–2.656	0.405	1.224	0.756–1.982	0.41
**BUN, mg/dL**	0.992	0.974–1.01	0.375	0.997	0.986–1.007	0.536
**Cr, mg/dL**	0.924	0.815–1.048	0.219	0.978	0.914–1.046	0.512
**Albumin, g/dL**	0.69	0.432–1.102	0.12	0.803	0.569–1.131	0.209
**Platelet count**	0.989	0.978–1	0.057	0.995	0.985–1.005	0.30
**Haematocrit, %**	0.997	0.964–1.031	0.853	0.995	0.973–1.018	0.673
**RDW day 1, %**	1.125	0.928–1.363	0.232	1.075	0.938–1.232	0.30
**WBC day 1**	0.951	0.895–1.010	0.102	1.020	0.952–1.056	0.263
**DNI day 1, %**	1.049	1.02–1.08	0.001*	1.041	1.016–1.066	0.001*
**DNI day 2, %**	1.036	1.015–1.057	<0.001*	1.021	1.003–1.039	0.023*
**DNI day 3, %**	1.082	1.042–1.124	<0.001*	1.026	0.998–1.055	0.072
**DNI day peak, (%)**	1.031	1.01–1.052	0.004*	1.012	0.996–1.029	0.142
**DNI peak, day (day)**	1.046	0.977–1.12	0.196	1.01	0.942–1.082	0.787
**Antibiotics prophylaxis**	0.568	0.174–1.852	0.3485	0.966	0.482–1.938	0.9235
**Inotrophics**	0.859	0.264–2.792	0.8	1.055	0.559–1.991	0.8686
**APACHE II**	1.01	0.957–1.066	0.7183	0.998	0.962–1.036	0.9277

CPC: cerebral performance category, ACS: acute coronary syndrome, ROSC: return of spontaneous circulation, CPR: cardiopulmonary resuscitation, BUN: blood urea nitrogen, Cr: creatinine, RDW: red cell distribution width, APACHE II: Acute Physiology and Chronic Health Evaluation II, DNI: delta neutrophil index

*: *p*<0.05.

**Table 3:** Multivariate Cox proportional hazard model for 30-day mortality and neurologic outcomes.

**30-day Mortality**		**Demographic data+DNI day 1**			**Demographic data+DNI day 2**			**Demographic data+DNI day 3**			**Demographic data+DNI peak**	
**Variables**	**HR**	**95% CI**	**p-value**	**HR**	**95% CI**	**p-value**	**HR**	**95% CI**	**p-value**	**HR**	**95% CI**	**p-value**
**Age**	1.025	1.-1.051	0.051	1.020	0.995–1.047	0.124	1.019	0.984–1.056	0.289*	1.023	0.992–1.055	0.140
**Sex (female)**	1.320	0.618–2.821	0.473	1.484	0.677–3.253	0.324	0.976	0.357–2.667	0.962	0.697	0.253–1.919	0.485
**CPR rhythm (shockable)**	0.507	0.189–1.360	0.177	0.449	0.162–1.246	0.124	0.638	0.169–2.405	0.507	0.489	0.136–1.754	0.272
**DNI day 1**	1.055	1.023–1.087	<0.001*									
**DNI day 2**				1.040	1.018–1.064	<0.001*						
**DNI day 3**							1.079	1.038–1.121	<0001*			
**DNI peak**										1.028	1.006–1.052	0.015*
**30-day CPC**		**Demographic data+DNI day 1**			**Demographic data+DNI day 2**			**Demographic data +DNI day 3**			**Demographic data +DNI peak**	
**Variables**	**HR**	**95% CI**	**p-value**	**HR**	**95% CI**	**p-value**	**HR**	**95% CI**	**p-value**	**HR**	**95% CI**	**p-value**
**Age**	1.025	1.008–1.043	0.004*	1.026	1.008–1.0445	0.004	1.023	1.003–1.043	0.022	1.025	1.005–1.044	0.013*
**Sex (female)**	0.901	0.513–1.581	0.719	0.889	0.505–1.566	0.683	0.761	0.402–1.440	0.401	0.684	0.358–1.307	0.250
**CPR rhythm (shockable)**	0.734	0.393–1.377	0.333	0.749	0.398–1.410	0.371	0.886	0.438–1.794	0.737	0.776	0.380–1.584	0.486
**DNI day 1**	1.040	1.014–1.067	0.002*									
**DNI day 2**				1.021	1.003–1.039	0.023*						
**DNI day 3**							1.024	0.995–1.054	0.106			
**DNI peak**										1.011	0.995–1.028	0.188

*: *p*<0.05.

**Fig 1:**
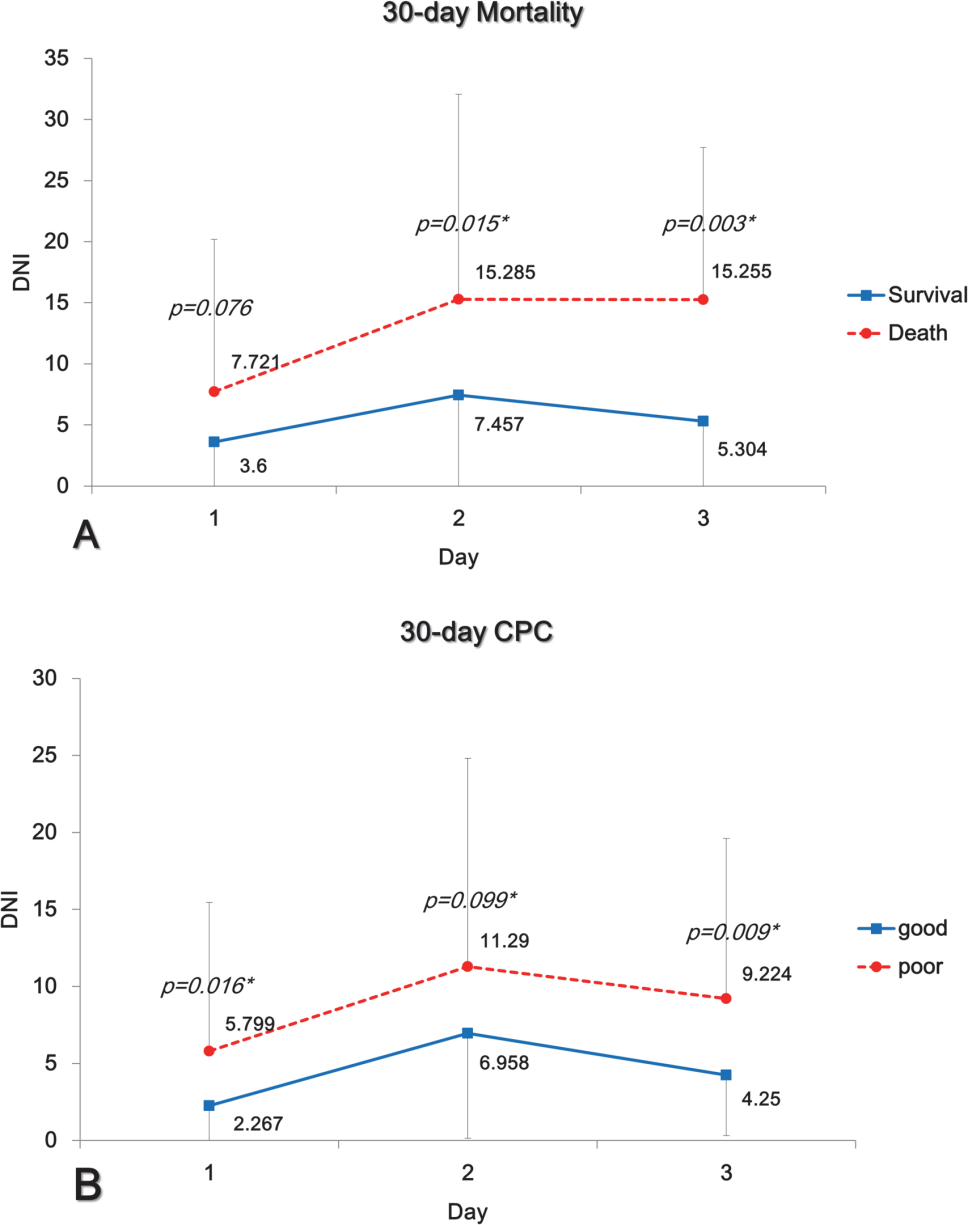
Mean delta neutrophil index (DNI) values associated with mortality (A) and neurologic outcomes (B) at 30 days. Days: Days after out-of-hospital cardiac arrest.

**Fig 2:**
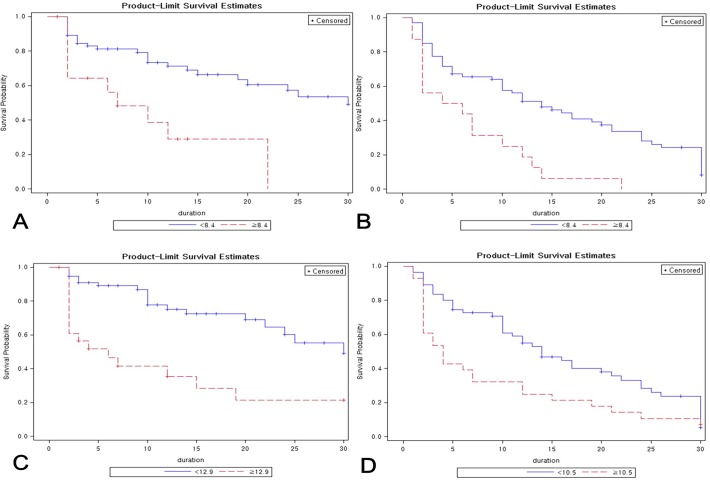
Delta neutrophil index as a predictor of mortality and neurologic outcomes at 30 days after out-of-hospital cardiac arrest. The technique of Contal and O’Quigley based on the log-rank test was used to assess prognostic value of the delta neutrophil index (DNI) by considering time to event. Cut-off points for DNI measured on day 1 were (A) DNI >8.4 (p = 0.01), HR = 3.227 (95% CI, 1.495–6.967; p = 0.001) for 30-day mortality and (B) DNI >8.4 (p = 0.01) HR = 2.718 (95% CI, 1.508–4.899; p<0.001) for neurologic outcomes at 30 days. Cut-off points for DNI measured on day 2 were (C) DNI >12.9 (p<0.001), HR = 3.292 (95% CI, 1.662–6.519; p<0.001) for 30-day mortality and (D) DNI >10.5 (p = 0.004), HR = 1.709 (1.051–2.778; p = 0.02) for neurologic outcomes at 30 days. Duration: Days after out-of-hospital cardiac arrest.

Mean DNI values differed significantly between groups with respect to 30-day mortality and neurologic outcomes (Fig. 1). Kaplan–Meier curves were generated for 30-day mortality and neurologic outcomes based on DNI values on day 1 and 2, and results of the log-rank test indicated that these DNI values were independent prognostic factors in OHCA (Fig. 2). Optimal day 1 DNI cut-off values were 8.4% (p = 0.01) for 30-day mortality and 8.4% (p = 0.01) for 30-day neurologic outcomes, and optimal day 2 DNI cut-off values were 12.9% (p<0.001) for 30-day mortality and 10.5% (p = 0.004) for 30-day neurologic outcomes.

Taken together, results of the Contal and O’Quigley technique, Kaplan–Meier analysis, and log-rank test by considering time to event demonstrated that increased 30-day mortality in patients with OHCA was associated with DNI >8.4% on day 1 (HR, 3.227; 95% CI, 1.485–6.967; p = 0.001) and >12.9% on day 2 (HR, 3.292; 95% CI, 1.662–6.519; p<0.001). In addition, poor neurologic outcomes were associated with DNI >8.4% on day 1 (HR, 2.718; 95% CI, 1.508–4.899; p<0.001) and >10.5% on day 2 (HR, 1.709; 95% CI, 1.051–2,778; p = 0.02).

## Discussion

In this study we found that DNI in the early post-resuscitation phase after OHCA could be an independent predictor of 30-day mortality and neurologic outcomes. Our results suggest that patients with ROSC after cardiac arrest should be carefully monitored if the DNI value exceeds 8.4%. Similarly, a previous study by Park et al. reported that DNI >6.5% was a good diagnostic marker of severe sepsis and septic shock within the first 24 hours after intensive care unit admission.[10] In our study, the cut-off value for DNI to predict mortality in OHCA was higher than the value reported in other critical settings like severe sepsis or septic shock. After post-resuscitation, most patients may present with a more severe clinical course compared to patients with severe sepsis or septic shock.

Nolan et al. proposed post-cardiac arrest syndrome as a second, more complex phase of resuscitation in patients who achieved ROSC after cardiac arrest.[5,6] Both infectious and sterile injuries (e.g., ischaemia/reperfusion) initiate and propagate the systemic inflammatory response and organ damage. [13] During infection or stress, immature neutrophil forms enter the circulation. The elevated immature/total granulocyte ratio, referred to as a left-shift,[10] is therefore a marker of infection or systemic inflammation. An increase in immature granulocytes is also included in the criteria of systemic inflammatory response syndrome (SIRS). [7] Previous studies have described DNI as a useful prognostic factor for severity of sepsis and septic shock, positive blood culture results, disseminated intravascular coagulation, and mortality.[8,9,10] Recent studies suggest that sepsis is able to impair innate immunity.[7,10] However, it is unclear how sterile injury activates innate immunity in the absence of foreign invaders.[5,6]

Whole-body ischaemia/reperfusion after cardiac arrest activates immunological and coagulation pathways, increasing the risk of infection and multiple organ failure. In the early phase (within 3 h after cardiac arrest), blood levels of cytokines, soluble receptors, and endotoxin are elevated. These changes are associated with clinically poor outcomes.[5,6] In sepsis, the overproduction of nitric oxide (NO), chemokines, and cytokines reduces chemotaxis and adhesion interactions between neutrophils and the endothelium. Alves-Filho *et al*. suggest neutrophil paralysis to be characterized by the failure of neutrophils to migrate into the site of infection, and inappropriate neutrophil sequestration in remote organs.[14]

We believe that post-cardiac arrest syndrome is comparable with sepsis or SIRS. In post-cardiac arrest syndrome, elevated P- and E-selectin, soluble vascular cell adhesion molecule-1, and soluble intercellular molecule-1 are observed after CPR.[6,15] Elevated adhesion molecules indicate neutrophil activation and subsequent endothelial injury.[6,15] The hyporesponsiveness of circulating leukocytes, like neutrophil paralysis in patients with systemic inflammatory response syndrome, appears to protect against the overwhelming pro-inflammatory response.[6,15]

Although the mechanisms underlying the increase in immature granulocytes after post-resuscitation are unclear, we hypothesize that neutrophil paralysis in post-cardiac arrest syndrome leads to interactions between adhesion molecules and activated neutrophils causing decreased circulating neutrophils. The subsequent hyporesponsiveness of circulating leukocytes may require more activated neutrophils after ischaemia/reperfusion injury. Therefore, levels of immature neutrophils may be increased by the requirement for circulating activated neutrophils after post-resuscitation. It is unlikely that the elevated DNI in the early post-resuscitation phase is the result of infection because the DNI value on day 1 was determined from blood drawn before or immediately after ROSC. Therefore, the observed elevation in DNI is likely the result of sterile inflammation.

Procalcitonin and C-reactive protein have been widely used as biomarkers in critical care.[11,16] Although C-reactive protein and procalcitonin can increase the likelihood of a sepsis diagnosis,[17] Procalcitonin is associated with poor long-term neurologic outcomes and mortality in patients achieving ROSC after OHCA; however, C-reactive protein was not associated with outcomes.[16] In addition, elevated levels of procalcitonin and C-reactive protein do not correlate with infection in the early stage of post-cardiac arrest syndrome.[16]

In this study we used an automated blood cell analyser to determine DNI, which overcomes the limitation of manual counting of immature granulocytes. The immature granulocyte counts may be inconsistent with the results from other analyzers that use different methods to determine differential leukocyte counts. Ha et al. demonstrated that an elevated immature granulocyte % (IG%) of more than 2.0–3.0% in the Sysmex system could be detected for sepsis using different methods. [18] However, that study revealed that IG% could not estimate the severity of sepsis due to the inability of this method to predict 28-day mortality. [18]

While immature granulocytes have been measured using various equipment and statistical approaches, the majority of published studies use ADVIA to measure immature granulocytes.[18] DNI value has been accurately measured using a simple two-step procedure and staining of cytochemical myeloperoxidase compared to other analyzers. Firstly, this ADVIA analyzer analyzes the leukocyte differentials by cytochemical myeloperoxidase (MPO) reaction. Next, this machine confirms the nuclear lobularity of white blood cells by a light beam for accurate fraction of immature granulocytes in circulating blood. [9] Nahm et al. clearly demonstrated in using this approach, DNI strongly correlated with manual immature granulocyte counts. [9] Therefore, the assessing DNI using the ADVIA device may best reflect immature granulocyte counts compared to all available analyzers.

In addition, DNI can be analysed along with the complete blood count, which is routinely performed in critically ill patients,[8] requiring no additional time or cost unlike C-reactive protein and procalcitonin. Changes in DNI over time may also be promising marker for the effects for therapeutic hypothermia, and Pyo et al. demonstrated that DNI could discriminate between a disease flare-up and infection in systemic lupus erythematous [19] and we proposed that DNI may be promising marker for development of sepsis during post-resuscitation care. In future, prospective multicentre studies with a larger number of patients will be needed to confirm our findings that DNI is a promising marker prediction of prognosis after ROSC and discrimination between severe sepsis and post-cardiac arrest syndrome during post-resuscitation care.

### Study limitations

This study has several limitations. First, this was a single-centre retrospective study, raising the possibility of selection bias. Second, the sample size was relatively small because we included only patients who survived at least 24 h and had at least two serum DNI measurements in the acute phase (24–48 h), and the rates of ROSC and survival after OHCA are lower in our country than in developed countries. However, statistically, repeated measurements of DNI over time in one subject could improve statistical power and compensate for the small sample size in this study. Third, this study did not evaluate haemodynamic parameters, presence of infection, or organ function to investigate the mechanisms underlying changes in DNI in the early post-resuscitation phase. Fourth, although our study revealed that DNI in the early post-resuscitation phase was associated with 30-day mortality and neurologic outcomes, we were unable to assess long-term neurologic outcomes in OHCA patients. In conclusion, a higher DNI is a promising prognostic marker for 30-day mortality and neurologic outcomes after OHCA. Patients with elevated DNI on day 1 and day 2 after OHCA might be closely monitored so that appropriate treatment strategies can be implemented.
